# Proposition to Establish a Lectureship on Dental Surgery in Medical Colleges

**Published:** 1851-06

**Authors:** E. B. Gardette


"*Proposition to establish a Lectureship on Dental Surgery in Medical Colleges.*"

The suggestions published under this head, have, as I might naturally expect, attracted the notice and met the condemnation of the "Journal of Dental Science," whose senior Editor is the "Professor of Principles and Practice" in the Baltimore College of Dental Surgery; and while I desire that the merits or value of my proposition, to the community and to the profession, may be fairly judged by the parties truly concerned in its utility or otherwise, I design to take only such part in the discussions it may provoke, as seems necessary to make myself understood. This duty performed, I leave the proposal, be it what it may, to take care of itself, with no more vanity, interest, or ambition about its adoption than becomes a plain man, when he honestly contributes his thoughts with the hope of being useful.

I imagined that any one at all familiar with the subject, on reading the paper referred to, would perceive that it did not attempt to set forth *the manner* in which the plan is to be carried out; justly leaving this to be taken into consideration by Trustees and Medical Faculties—"*in such form as may seem most proper to their own judgments.*" But the absence of details or "reasons" from me, by which to prove the practicability of my proposition, appears to be considered by the Professor as *prima facie* evidence that it is impracticable. It is very true I did not deem it necessary to give reasons "*showing that Dental Surgery could be taught by an adjunct Professor equally as well as by five instructors, who are now found necessary*:" it will be for the Trustees of medical colleges to determine these questions, should they see fit to inquire through the medium of competent and disinterested testimony, how far the proposed Lectureship would answer the objects contemplated, or whether it would be advantageous to the public, and consistent with the best medical and dental education.

There is no language of mine from which the Professor could rightly infer the thoughts he attributed to me, when he says:—"*Yet Dr. Gardette thinks that all this work can be done equally as well by an adjunct Professor, who shall be attached to the Medical Faculty, lecturing when they will give him an opportunity—to students when they will give him a hearing.*" I neither think nor expressed any such absurdity; the *work* to be done by a dental student I said nothing about, but am of opinion it should be executed in the office or laboratory, and under the superintendance of his private preceptor, and it is there that careful, judicious operators will be made, whether they listen to Lectures in Medical or Dental Colleges.

I have suggested no scheme "*according*" *to which all the medical students must be instructed in dentistry, nor have I said that* "*dental students are to be thoroughly instructed in all the branches of medicine.*" The abstracted Professor, I must suppose, imagines these statements, (I certainly did not make them,) for the mere sake of pointing out their erroneous character; and by means of these suppositious facts he makes for me the following arithmetical calculation:—"*In four months he would lecture about a hundred times; or during a hundred hours, being divided*

*into working days, would give ten days entire to dentistry; in which time all necessary information is to be imparted—all necessary skill acquired.*" To attribute opinions so perfectly and ridiculously ignorant to me, seems anything but consistent with the expressions of regard or just claims to respect, with which the Professor opens his review. I am at a loss to explain so absolute an incongruity, unless by supposing that my "*strange and startling proposition*" produced consternation in the Professor's mind, in the midst of which he seems to have mistaken me for a Dental College; a singular mistake, but which affords the only mode of interpreting the term "*suicidal*" which he applies to my proposition, reproaching me with making it "*at a most unfortunate time*" for Dental Colleges, and endeavoring to show that its influence was fatal to *their* growing prospects.

Now with reference to the supposed injury done to Dental Colleges by my suggestions, the Professor indulges in long lamentations about the "*past degradation*" of the Dentist's profession—his "*sullen and fretful isolation*"—when "*all was dark and hopeless.*" In these strains, my good friend is more poetical than precise or historical; and I cannot subscribe to this and much similar clamor, by which one is to suppose that Dentists and Dental Surgery were good for nothing at all—had no claim to sympathy or respect, until ten or twelve years ago when a Dental College came into existence. The Professor-Editor seems to forget the lives and labors of valuable men, some of whom are yet among us, while the memories of others are justly cherished by their fellow men, to whom we owe the progress of Dental Surgery in the last forty or fifty years; it is in vain, if not vanity, to suppose that such professional improvement is the spontaneous growth of the present time, instead of a gradual consequence of individual mind, skill, and example, belonging to the past. Even our humble selves—you and I, my dear Doctor—have done some service in the march of improvement, without having had the benefit of either Lectures (except medical ones) or Dental Colleges: and there are several names we could mention of past and present times, who, without such advantages, have done honor to their profession and the communities of which they were members.

The very term "*profession*," according to the Professor, would seem to have had no existence or application to the Dentist, except through the medium of *Professors;* he says, "*at this period, when all was dark and hopeless,* (12 years ago!) the idea was conceived of establishing a Dental College, and '*professionalizing*' Dental Surgery." . . . . . . "A beginning had been made. *A profession of Dental Surgery,* standing upon its own merits, respected for its own sake, had *been recognized* in the country." The Professor cannot surely and seriously mean to say that the establishment of a Dental College was the *beginning* of Dental Surgery! that the occupation of the Dentist was not "*professionalized,*" or recognized as a *profession,* until embodied and represented by a Professor Dentist in a Dental College!

But to return to the suggested change in that portion of a Dentist's education which may be advantageously pursued in a public school, and which I have taken the liberty to think can be better accomplished through Lectureships in Medical than in Dental Colleges.

To set forth the details and principles that are to govern in any new system or proposed improvement in professional education, is an important and responsible duty; and ere such a suggestion is acted upon by the Trustees of prominent Medical Colleges, they will no doubt carefully examine into the objects and practicability of the measure. With the aid of their Medical Faculty, and men having knowledge and experience as Dentists, they would establish such a curriculum of studies, as would insure the best results; and will not overlook the fact that private instruction in the office of a Dentist of standing, during a period specified (as is required of the medical student) should be among the conditions for graduating.

The Professor is right in supposing I did "*not contemplate that any instruction in practical Dentistry should be given in the Medical Colleges,*" and of course it was from private teaching by a preceptor that I expected this "*essential part of a Dentist's education*" would be obtained; for I think this the *only* mode in which it can be acquired with that degree of judgment and safe manipulation which will command confidence. And yet I can see no good reason why the surgical clinics of our Medical Colleges, (in the event of a Lectureship on Dentistry) should not embrace many operations on the living teeth. Mechanical Dentistry—or making artificial teeth, seems the only branch excluded of necessity, from Medical Colleges, and this would not be undergoing any great *descent*, as the Professor well knows, should it continue chiefly in the goldsmith's shop.

I can foresee none of the calamitous results to medical character or Dentistry predicted by the learned Professor, in the event of Lectureships on Dental Surgery in Medical Colleges; but on the contrary, I firmly believe that such a connection would tend to elevate the Dentist and his profession, benefit the practice of medical men under many circumstances, and advance the interests of suffering humanity. That many practicing Dentists in our country, are of this opinion, and are not satisfied with the establishment of separate Colleges for Dental Surgery, may naturally be inferred from the complaint of the Professor, that "*If half the students of Dentistry now in the offices of Dentists, should be sent to the Dental Colleges, then would they be able to take rank,*" *&c.*

It is common with Dentists of high standing, to require of their pupils that they attend medical lectures, and take the degree of M. D.; and this is the case *even since the existence of Dental Colleges.* It may therefore be reasonable to suppose that the Lectureships suggested in Medical Colleges would meet the approbation of the best practising Dentists of the country, and that their students would gladly attend such lectures. I cannot see the wide difference that would exist between C. A. Harris, M. D., Professor of the Principles and Practice of Dental Surgery, in the Washington Medical College, or the University of Pennsylvania, and C. A. Harris, M. D., Professor of Principles and Practice, in the Baltimore College of Dental Surgery. The learning, the industry and eloquence would be the same in both positions; but I think these qualifications would be displayed in the former case to the students *from all the Dentists' offices*, while in the latter, after "*twelve years*" of uphill work, "*endurance and toil*," he complains that "*very few of the educated class of Dentists encourage the undertaking, while very many deride it, and use all their influence to keep students away.*"

The reproach of the Professor that my proposition has been "*made at a most unfortunate time*," is followed by an attempt to show that the Baltimore College had just succeeded; that

"*opposition was beginning to grow weaker—sympathy stronger,*" and that "*a few years would bring about a general ascent from degradation.*" So far as regards the time chosen for giving publicity to my proposition, the Professor is neither just or generous—for the probable influence of such a proposal, if it have any, upon the prosperity of the College, would certainly have been greater when it was in its infancy, than now. Sentiments of respect and kindness for gentlemen connected with, or interested in the first Dental College, and a willingness to see the scheme tried, prompted myself and others to lend it aid and not condemn precipitately.

It has been stated to me (and the Professor says, "*the truth must be told,*") that the Baltimore Dental College has been chiefly sustained thus far in its feeble existence by the talents and influence of its *medical* Professors, and that one of these, who has been the main prop of the Institution, has of late years designed to abandon it as an expensive unsuccessful attempt. This state of things, when added to the Professor's acknowledgment, that less than half the students in Dentists' offices would suffice to make the Dental Colleges prosper, and that in twelve years they had not obtained a sufficient share of that amount of patronage, would seem to render this a fit and proper time to suggest some plan of Dental education that will be more acceptable and more successful.

In conclusion I beg to remark, that I did not expect to be called upon to prove the *practicability* of my proposition; it was its propriety and expediency which I feared might not so readily be established in the minds of those who have thus far neglected the speciality of the Dentist. But if Dentistry is "*a regular branch of medicine,*" (as Professor Bond justly remarks) "*in which relation only it can be regarded as a scientific pursuit, and the practice of it esteemed a profession,*" then the proper place for having its principles taught, is a Medical College, and the proper title or diploma of its representatives should originate from the same source as do those of other branches of medical science. *Dental* College seems scarcely to be the correct name for an Institution mainly sustained and conducted by medical teachers, and until a Faculty can be formed entirely of Dentists, the title no less than the scheme is inappropriate.

The Baltimore Professor says that the plan of Lectureships on Dental Surgery in Medical Colleges "*is not a novel one*," because "*thirty-five years* ago the late Dr. Hayden "*delivered a course of Lectures on Dental Surgery in the University of Maryland, but the experiment was unsuccessful.*" I take no especial pride in the originality of my proposition, and if in reality a similar one was promulgated by so learned and distinguished a Dentist as the late Dr. Hayden, it is the more worthy of consideration from the Trustees of Medical Colleges.

E. B. Gardette.

Note.—Since writing the above, some one has kindly sent me a copy of the first number of the "New York Dental Recorder," in which my attention is called to a very sensible communication under the signature of "*A.*" and having for its title, "*An appeal to the Medical Profession in behalf of Dental Science.*" This paper embodies the same thoughts and embraces a plan of Dental education very much the same as that I propose; I had never before seen or heard of the article referred to, and in truth did not know the existence of the Dental Recorder so early in its career. I freely yield to the priority of the writer's suggestion, and only regret that his anonymous character, or the abandonment of his scheme, has denied it the importance and influence to which I think it justly entitled.

